# Supplementary material for: AAV-mediated long-term TBX18 expression causes cardiac fibrosis and fails to induce pacemaker activity in rodents
Source: J Clin Invest. 2026 Jun 11;136(13):e190632. doi: 10.1172/JCI190632 (PMC13318123; doi:10.1172/JCI190632)
Supplement: Supplemental data [file jci-136-190632-s053.pdf]

Supplemental material

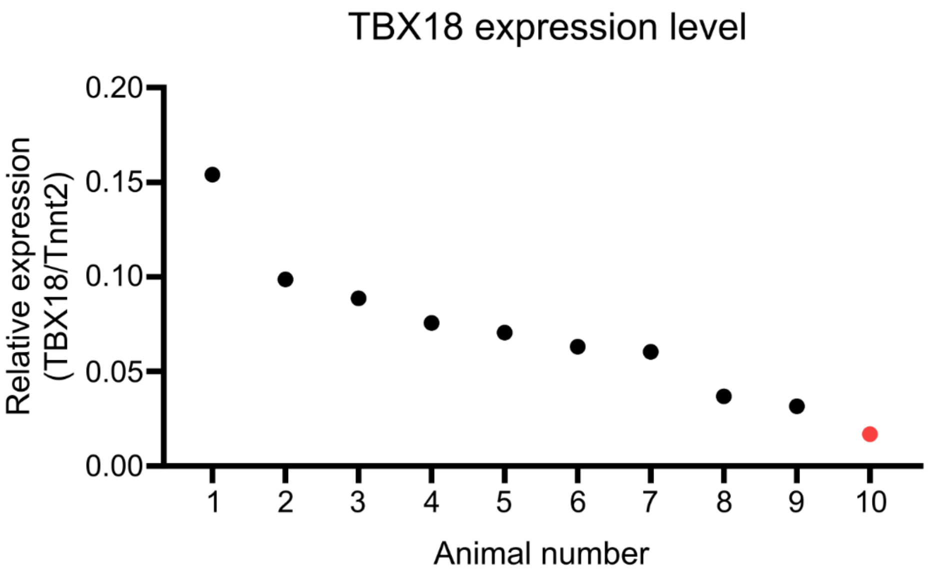

Supplemental Figure 1 TBX18 expression level in mouse hearts injected with AAV-TBX18 4 weeks post injection. Red dot indicates the single TBX18 heart in which no fibrosis was detected.

A

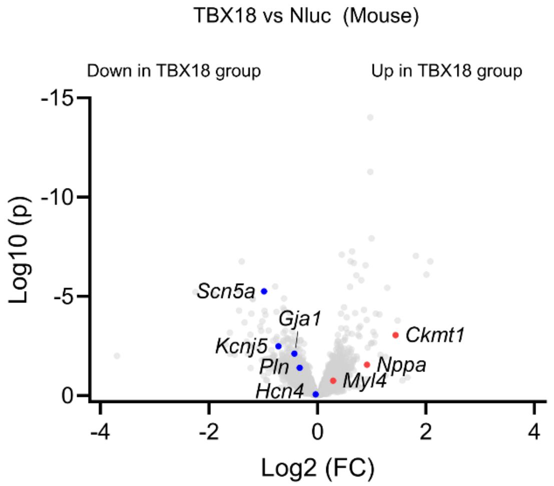

B

Enriched GO term in down-regulated genes	p-adjust
anatomical structure development (GO:0048856)	1.62E-10
developmental process (GO:0032502)	1.47E-09
cellular developmental process (GO:0048869)	5.83E-08
cell differentiation (GO:0030154)	7.69E-08
regulation of multicellular organismal process (GO:0051239)	3.39E-07
cell development (GO:0048468)	4.15E-07
multicellular organismal process (GO:0032501)	4.86E-07
monoatomic cation transmembrane transport (GO:0098655)	5.50E-07
inorganic ion transmembrane transport (GO:0098660)	1.33E-06
monoatomic ion transmembrane transport (GO:0034220)	1.52E-06

Enriched GO term in up-regulated genes	p-adjust
negative regulation of nitrogen compound metabolic process (GO:0051172)	1.35E-02
circulatory system process (GO:0003013)	1.44E-02
anatomical structure development (GO:0048856)	1.47E-02
developmental process (GO:0032502)	1.47E-02
negative regulation of cell communication (GO:0010648)	1.54E-02
sensory perception of smell (GO:0007608)	1.60E-02
cellular developmental process (GO:0048869)	1.65E-02
regulation of release of cytochrome c from mitochondria (GO:0090199)	1.68E-02
cellular process (GO:0009987)	1.69E-02
negative regulation of signaling (GO:0023057)	1.69E-02

Supplemental Figure 2 TBX18 expression in cardiomyocytes suppressed chamber myocardial genes.

(**A**) Volcano plot presenting gene changes between mouse hearts injected with AAV-cTnT-uORF-Nluc-GFP and AAV-cTnT-uORF-TBX18-GFP. (**B**) Gene ontology analysis on the most significantly up- and down-regulated genes in mouse hearts injected with AAV-cTnT-uORF-TBX18-GFP.

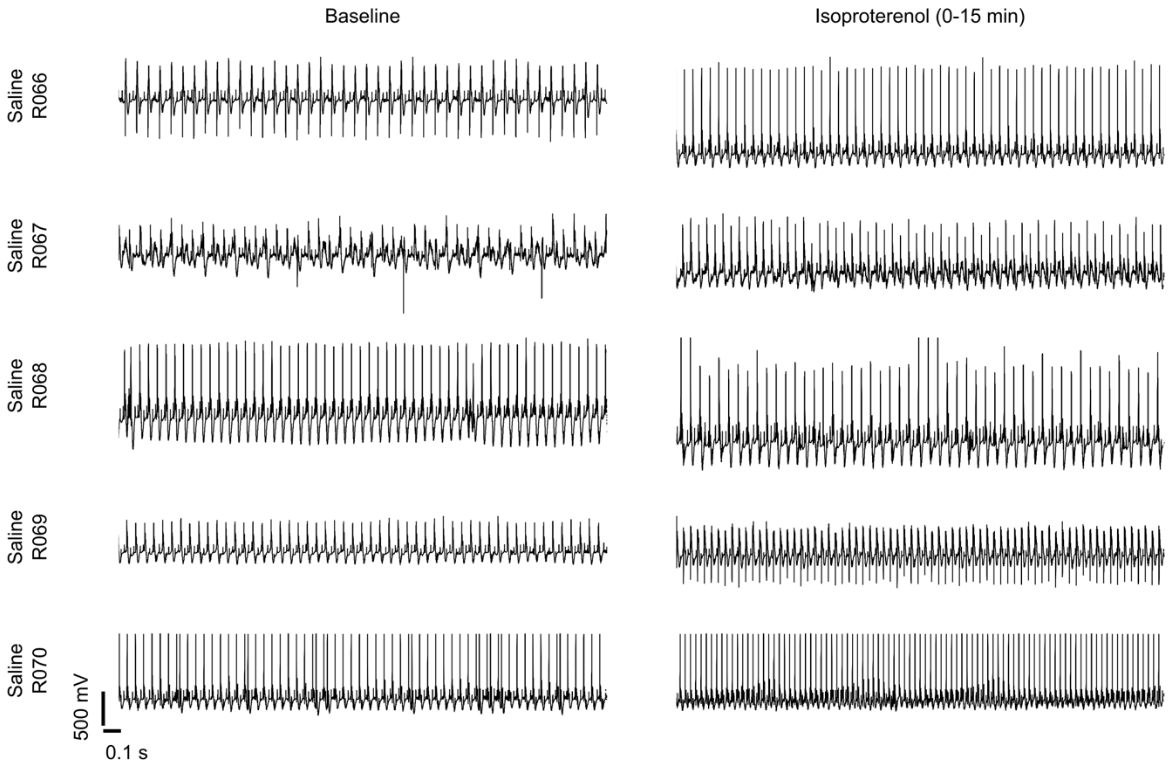

Supplemental Figure 3 ECG tracings of rats injected with saline at baseline (before the administration of isoproterenol) and 0-15 min after the administration of isoproterenol.

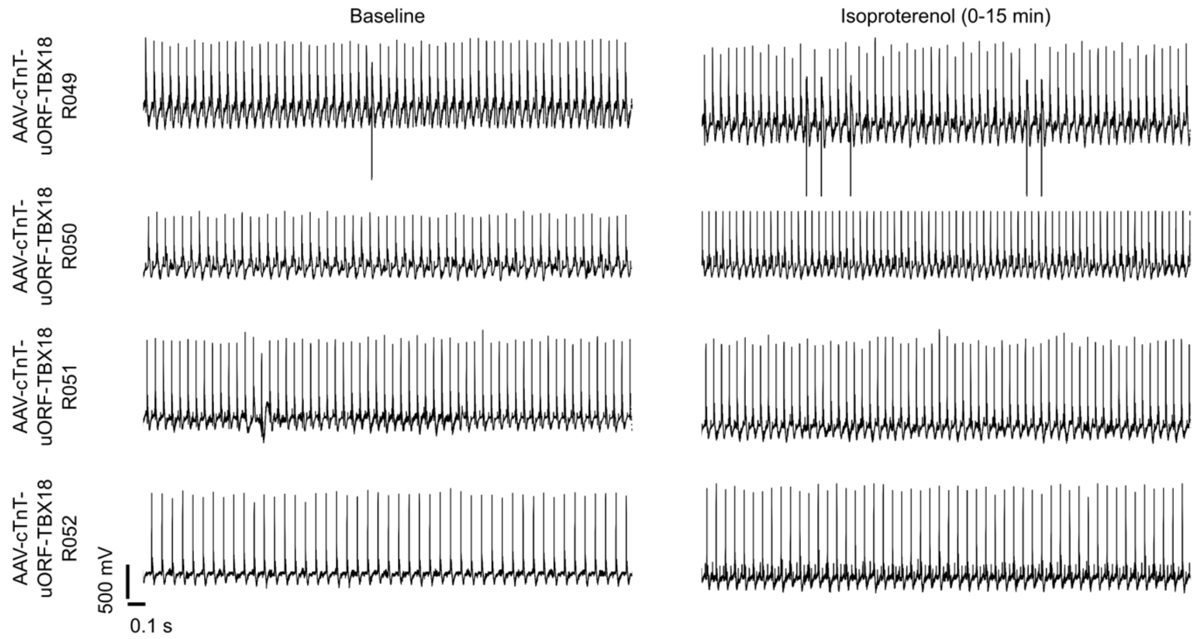

Supplemental Figure 4 ECG tracings of rats injected with AAV-cTnT-uORF-TBX18 at baseline and 0-15 min after the administration of isoproterenol.

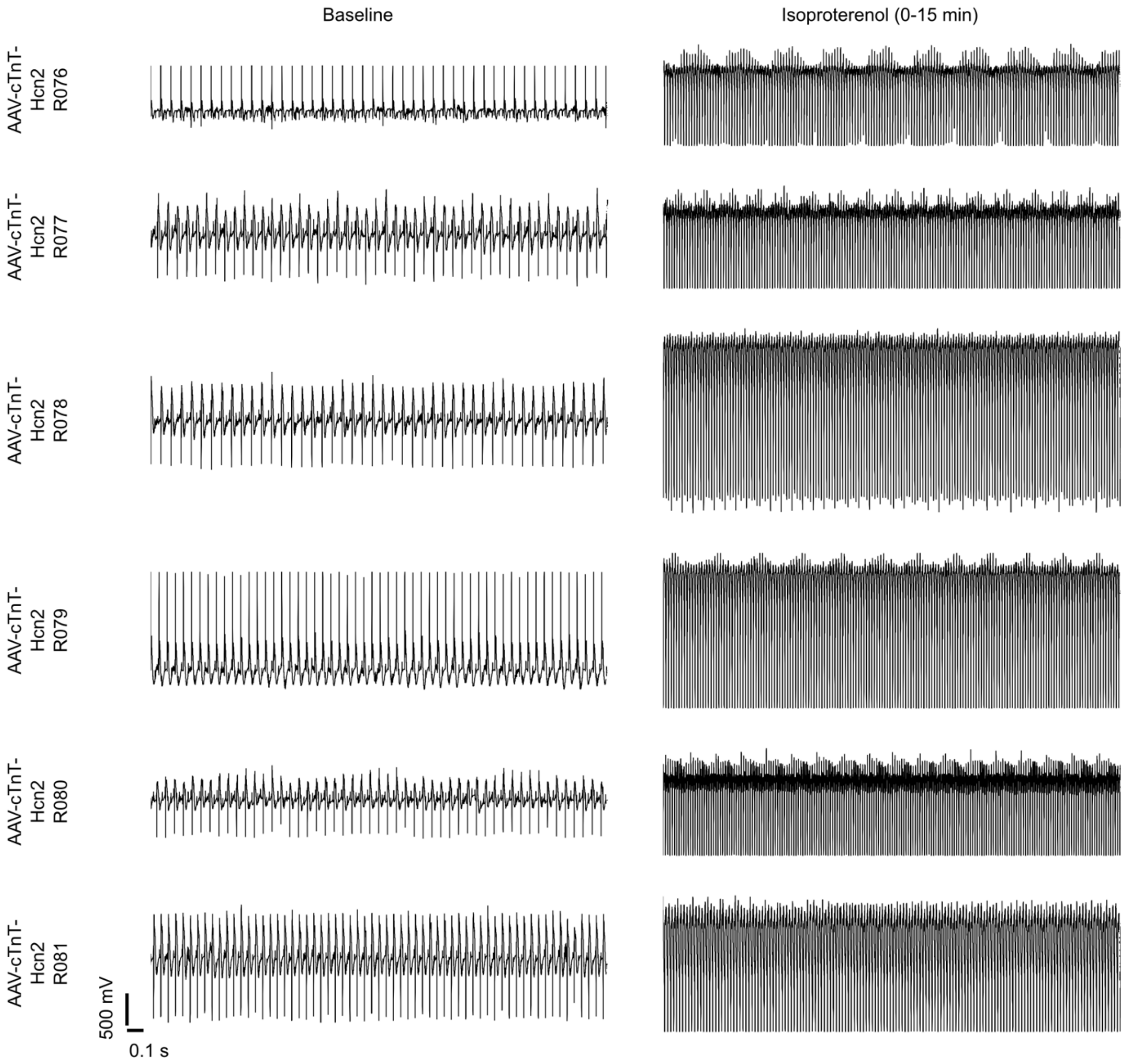

Supplemental Figure 5 ECG tracings of rats injected with AAV-cTnT-Hcn2 at baseline and 0-15 min after the administration of isoproterenol.

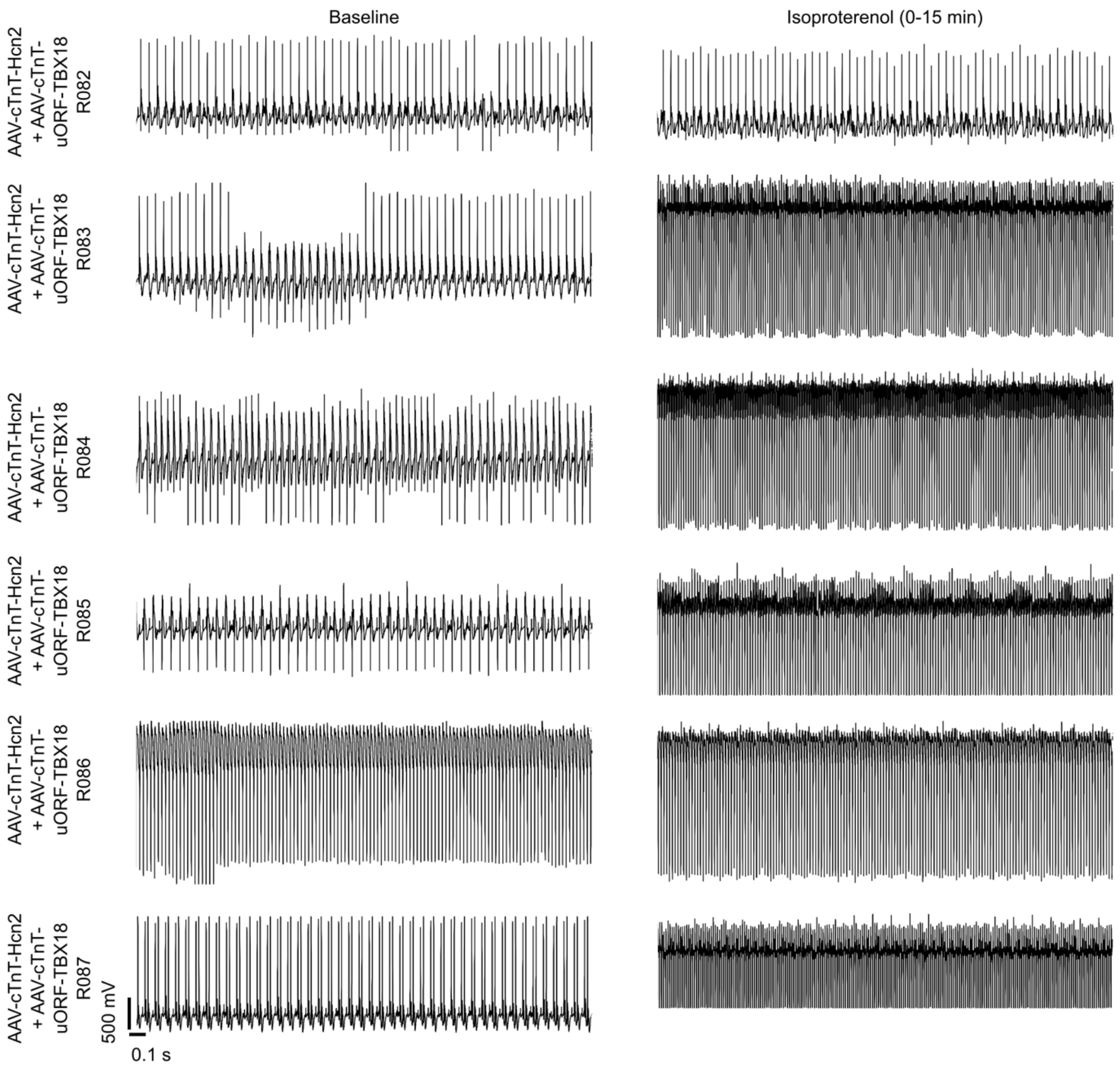

Supplemental Figure 6 ECG tracings of rats injected with both AAV-cTnT-Hcn2 and AAV-cTnT-uORF-TBX18 at baseline and 0-15 min after the administration of isoproterenol.

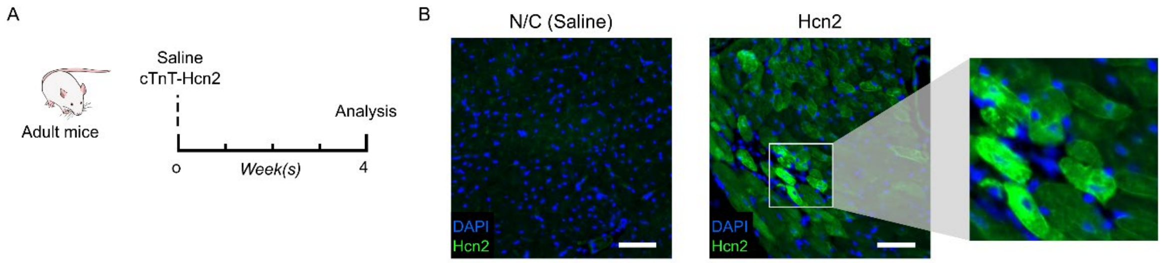

Supplemental Figure 7 Hcn2 is expressed in the left ventricle following AAV-cTnT-Hcn2 injection.

(**A**) Experimental design for histological analyses. (**B**) Immunofluorescence staining images of FLAG-tagged Hcn2 in saline- and AAV-cTnT-Hcn2-injected animals 4 weeks post injection. N = 3 for each group. Scale bar = 50 μm.

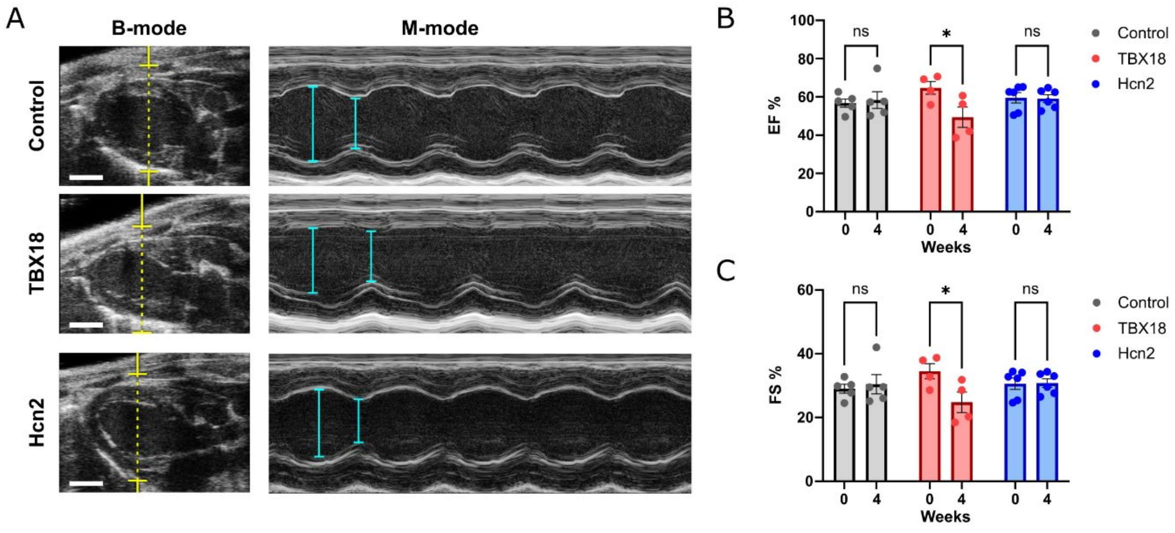

Supplemental Figure 8 AAV-mediated long-term Hcn2 expression does not impair cardiac function

(**A**) Representative echocardiographic images of B-mode (left) and M-mode (right) from mice injected with AAV-cTnT-control, AAV-cTnT-TBX18 or AAV-cTnT-Hcn2 4 weeks post injection. Yellow lines indicate the M-mode cut plane. Cyan lines indicate the left ventricular end-diastolic and end-systolic dimension. Scale bar = 2 mm. (**B**) Echocardiographic quantification of ejection fraction. (**C**) Echocardiographic quantification of fractional shortening. (B-C) n = 5 for control, n = 4 for TBX18 and n = 5 for Hcn2. Data are shown as mean ± SEM. Data were compared using two-way ANOVA with *post-hoc* Holm-Šídák test. *$p < 0.05$; ns, not significant.

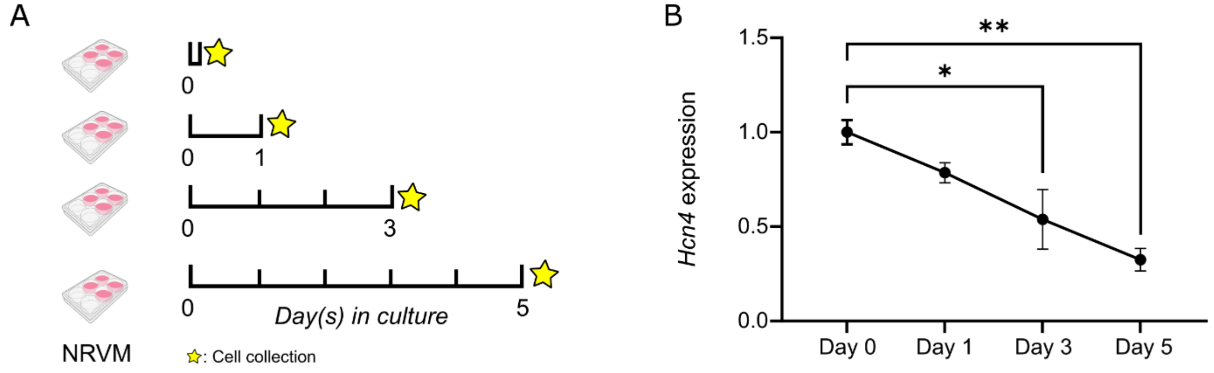

Supplemental Figure 9 *Hcn4* expression decreases over time in NRVMs.

(**A**) Experimental design. (**B**) Expression level of *Hcn4* in NRVMS at 0, 1, 3 and 5 days in culture. Data are shown as mean ± SEM. Data were compared using one-way ANOVA with *post-hoc* Holm-Šídák test. *p < 0.01; **p < 0.001.

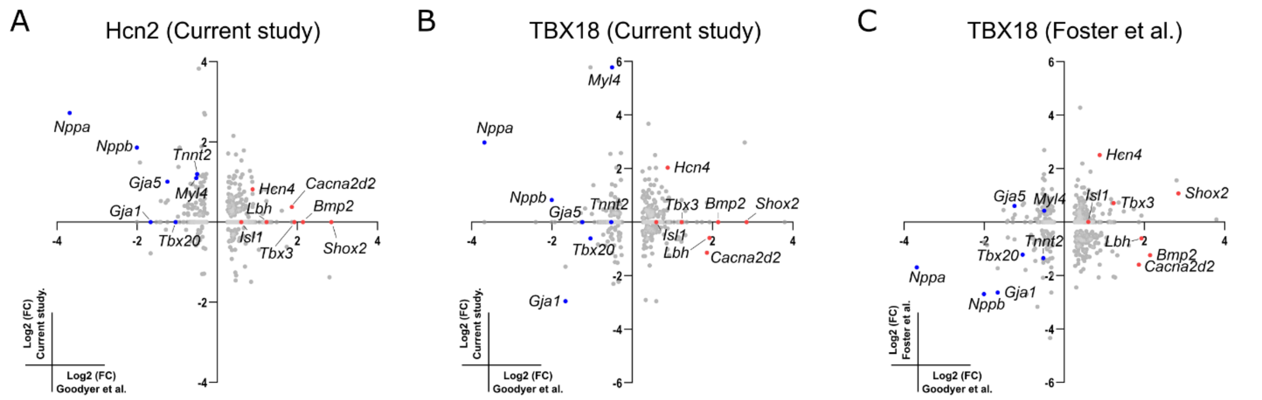

Supplemental Figure 10 Scatter plots showing the fold change of SAN marker genes selected from Goodyer et al. in various RNA-seq datasets.

(**A**) NRVM transduced with Hcn2 from current study. (**B**) NRVM transduced with TBX18 from the current study. (**C**) NRVM transduced with TBX18 from Foster et al.
