# Supplementary material for: AAV-mediated long-term TBX18 expression causes cardiac fibrosis and fails to induce pacemaker activity in rodents
Source: J Clin Invest. 2026 Jun 11;136(13):e190632. doi: 10.1172/JCI190632 (PMC13318123; doi:10.1172/JCI190632)

Full unedited blots for Figure 4E

Antibodies:
1st antibodies:
Rabbit-Anti-FLAG (Sigma, F7725, 1:500)
Mouse-Anti-b-Actin (Sigma, A5441, 1:5000)
2nd antibodies:
Sheep-Anti-Mouse (Sigma, NA9310; 1:5000)
Donkey-Anti-Rabbit (Sigma, NA9340, 1:5000)

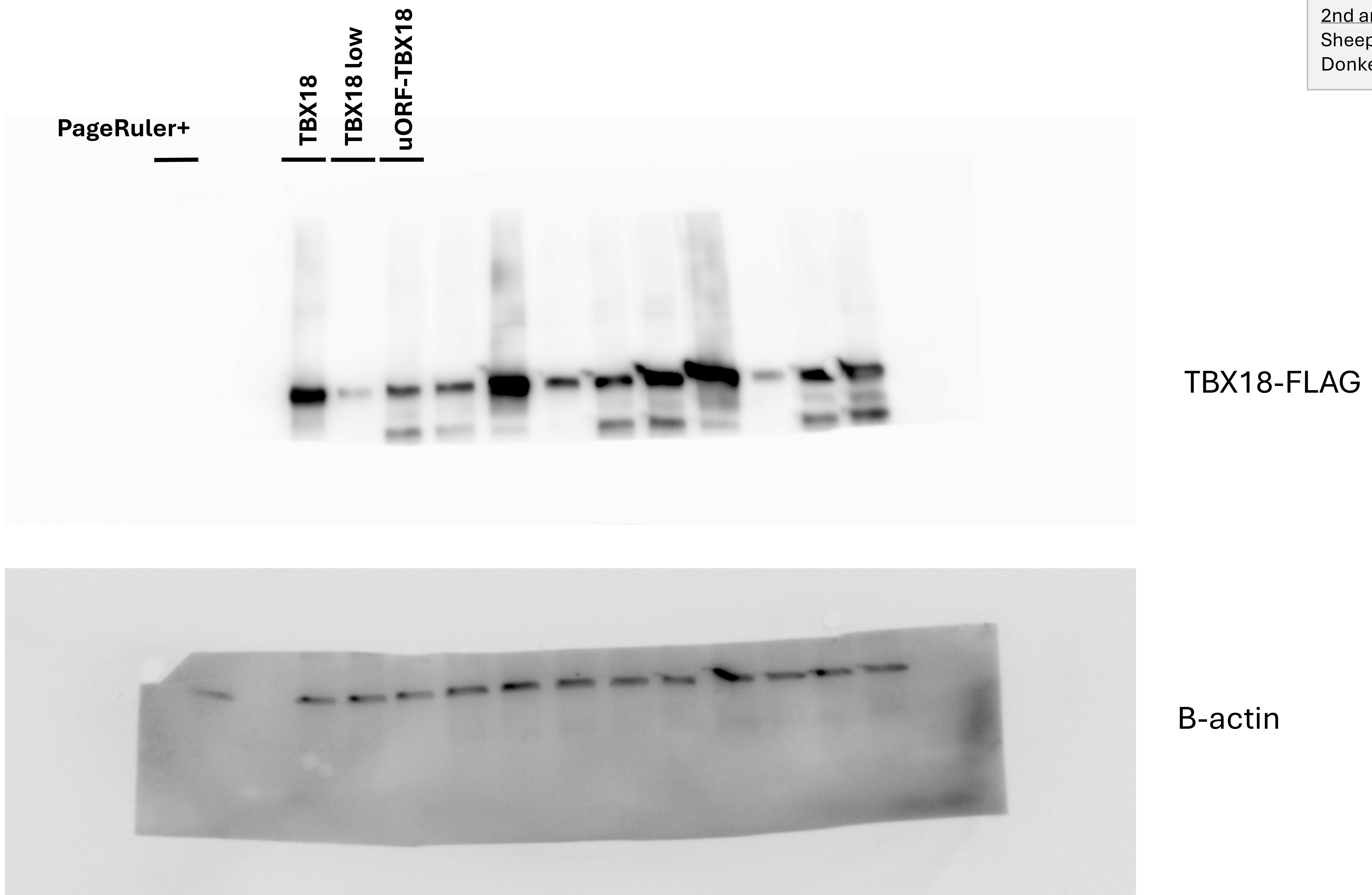

Full unedited blot for Figure 4G

Antibodies:
<u>1st antibodies:</u>
Rabbit-Anti-FLAG (Sigma, F7725, 1:500)
Mouse-Anti-b-Actin (Sigma, A5441, 1:5000)
<u>2nd antibodies:</u>
Sheep-Anti-Mouse (Sigma, NA9310; 1:5000)
Donkey-Anti-Rabbit (Sigma, NA9340, 1:5000)

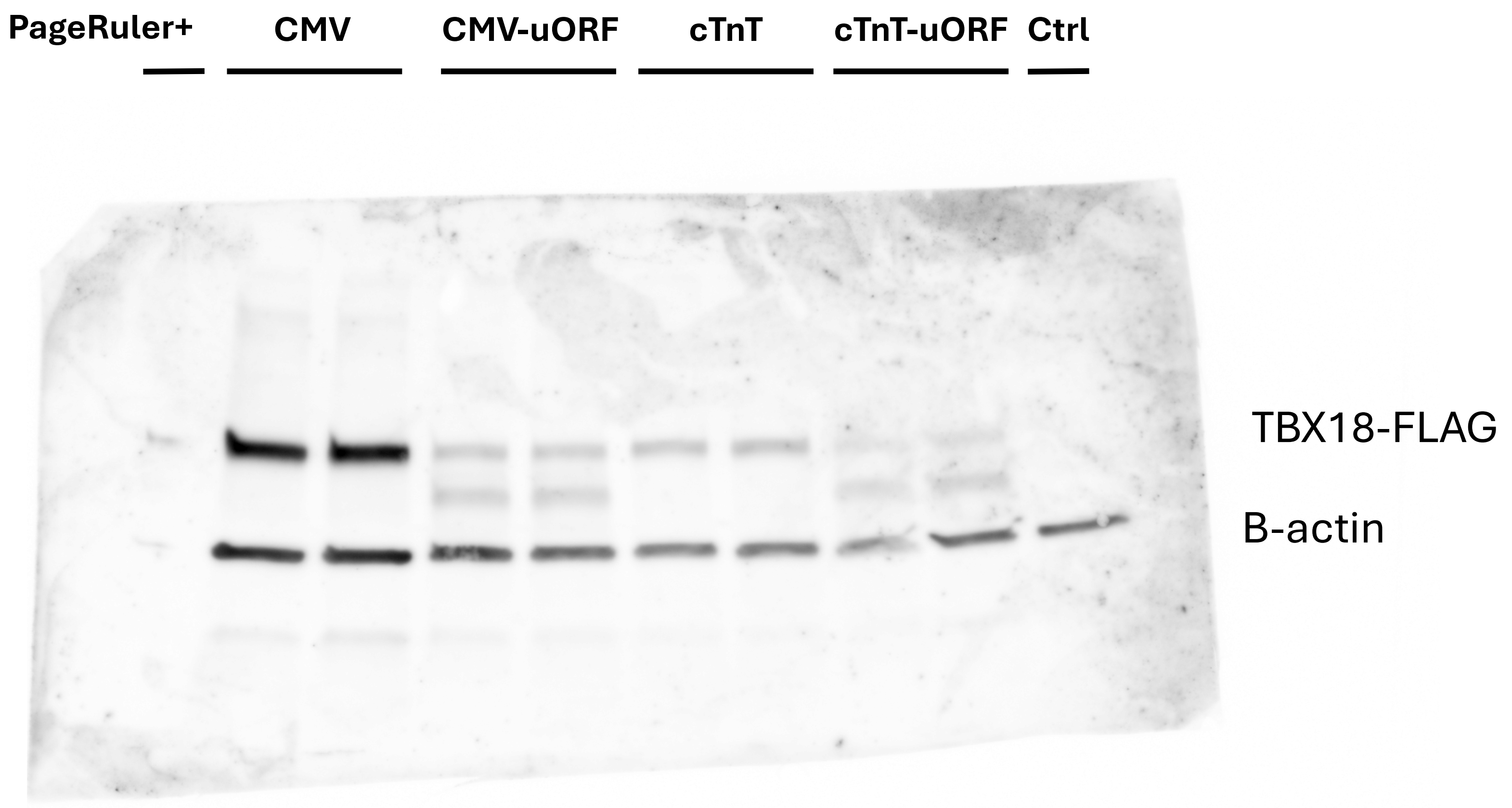

Supplement: Unedited blot and gel images [file jci-136-190632-s054.pdf]
